# Neutralizing Antibodies Targeting BK Polyomavirus: Clinical Importance and Therapeutic Potential for Kidney Transplant Recipients

**DOI:** 10.1681/ASN.0000000000000457

**Published:** 2024-07-09

**Authors:** Francois Helle, Aurélien Aubry, Virginie Morel, Véronique Descamps, Baptiste Demey, Etienne Brochot

**Affiliations:** 1 UR-UPJV4294, Agents Infectieux, Résistance et chimiothérapie (AGIR), Centre Universitaire de Recherche en Santé, Université de Picardie Jules Verne, Amiens, France; 2 Laboratoire de Virologie, Centre Hospitalier Universitaire, Amiens, France

**Keywords:** kidney transplantation, nephropathy

## Abstract

Most of the world's adult population is latently infected by the BK polyomavirus. It causes asymptomatic infection in healthy individuals but emerged as a threat to kidney transplant recipients because of virus-associated nephropathy caused by immunosuppressive therapy. In these conditions, when a functional cellular response is impaired by immunosuppression, neutralizing antibodies may play a major role because they can directly prevent infection of target cells, independently of cell-mediated immunity, by binding to the viral particles. Studying the contribution of anti-BK virus neutralizing antibodies in viral control has long been hampered by the lack of convenient *in vitro* models, but major progress has been made in the past decade. The four BK virus genotypes have been demonstrated to behave as distinct serotypes. A low recipient neutralizing antibody titer against the donor's serotype before kidney transplant has been significantly associated with BK virus replication after transplant. Different mechanisms exploited by the BK virus to evade neutralizing antibodies have been described. Recent studies also support the potential benefit of administering intravenous Igs or monoclonal neutralizing antibodies as a therapeutic strategy, and more interestingly, this could also be used as preventive or preemptive therapy before advanced kidney damage has occurred. Besides, neutralizing antibodies could be induced by vaccination. In this review, we summarize accumulated knowledge on anti-BK virus neutralizing antibodies as well as their clinical importance and therapeutic potential for kidney transplant recipients.

## Introduction

The BK virus is the first polyomavirus isolated in humans.^1^ BK virus primary infection likely occurs during early childhood. Then, the virus generally establishes a lifelong chronic infection in the urinary epithelium, with minimal clinical implications, although virions are periodically shed at low levels in urine of healthy people.^2,3^ However, the virus can cause serious complications in immunosuppressed individuals, such as nephropathy in 1%–10% of kidney transplant recipients, which can lead to graft failure in half of the recipients.^4^ Currently, no specific antiviral is commercially available for treating these conditions, mainly because polyomavirus replication does not involve viral enzymes.^5^ Consequently, kidney transplant recipients are managed by reducing the level of immunosuppressive therapy in response to the detection of high DNAuria or DNAemia (typically >7 and 4 log_10_ copies/ml, respectively), but this approach increases the risk of kidney transplant rejection.^6^ With the improvement of immunosuppressive therapy potency in the past few decades, BK virus thus emerged as a threat to kidney transplantation.

**Figure 1:**
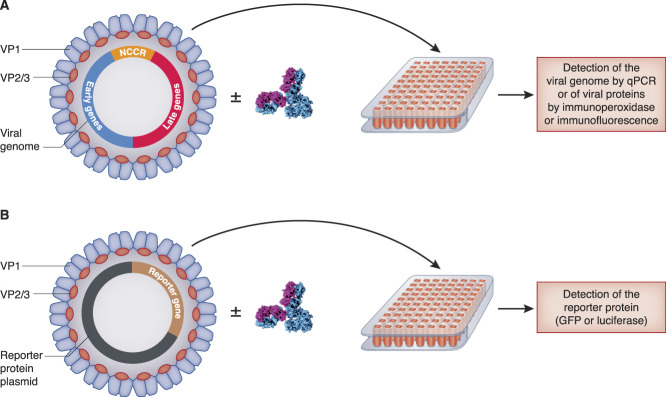
**Neutralization assays for BK virus.** (A) Naïve cells are inoculated with clinical isolate or recombinant virus (chimeric or not) in the presence or absence of serum/antibodies. The neutralizing activity is evaluated by comparing infection with and without antibodies, as determined by PCR, immunofluorescence, or immunoperoxidase. (B) Naïve cells are inoculated with pseudovirions representative of the different serotypes in the presence or absence of serum/antibodies. The neutralizing activity is evaluated by comparing infection with and without antibodies, as determined by detection of the reporter protein. GFP, green fluorescent protein; NCCR, non-coding control region; qPCR, quantitative PCR.

The BK virus genome is composed of two highly conserved regions coding for early and late proteins, separated by a noncoding control region.^5^ The early genes encode the large tumor antigen, small tumor antigen, and truncated tumor antigen that are involved in genome replication. The late genes encode the structural proteins VP1, VP2, and VP3 as well as the Agno protein, which are involved in virion assembly and release. BK virions are naked particles composed of 360 molecules of VP1 forming 72 pentamers residing on the outer surface, each pentamer interacting with a molecule of VP2 or VP3, residing in the inner part of the particles (Figure 1A).^7,8^

On the basis of nucleotide sequences, BK virus isolate genomes are categorized into four genotypes (I, II, III, and IV), two of which being divided into subtypes and subgroups (Ia, Ib1, Ib2, Ic, IVa1, IVa2, IVb1, IVb2, IVc1, and IVc2).^9^ PCR-based studies indicate that infection with BK virus genotype I is the most prevalent and distributed worldwide. The genotype IV only infects a minority of adults, ranging from 5% of clinical isolates in Sub-Saharan African individuals to 54% in Northeast Asian ones.^10^ Genotypes II and III are rarely observed.^11^ However, some studies raised the hypothesis that genotype II is more prone to clearance or have low reactivation potential as compared with genotype I and IV because its seroprevalence was higher than its PCR-based prevalence.^12–15^

Serum antibodies can be detected in more than 80% of the individuals by the age of 21 years, suggesting that most of the world's adult population is latently infected.^4^ Most of these antibodies are non-neutralizing and are directed at the removal of infected cells by antibody-dependent cell cytotoxicity, antibody-dependent cellular phagocytosis, or antibody-mediated complement-dependent cytotoxicity, all driven by their crystallizable fragments.^16^ Besides, neutralizing antibodies are of particular interest because they are able to inhibit the viral entry step independently of cell-mediated immunity. Through their antigen-binding fragment, these antibodies target VP1 that is the only protein accessible on the virion surface. Although the concentration of IgG in urine, and presumably the glomerular filtrate, is at least 1000-fold lower than that in serum of healthy adults,^17^ neutralizing antibodies may play an important role during transplantation, when a functional cellular response is impaired by immunosuppression.^18^ Indeed, using an *in vitro* polarized human renal epithelial cell model, BK virus has been suggested to spread intraluminally along the nephron to the pelvis and bladder *in vivo* and, interestingly, BK virus–specific antibodies could undergo transepithelial transport to inhibit *de novo* BK virus infection.^19^

Studying the contribution of neutralizing antibodies to BK virus control or reactivation has long been hampered by the lack of convenient *in vitro* models for evaluating the neutralizing activity of anti-BK virus antibodies. However, BK pseudovirions now enable easy, robust, and sensitive neutralization assays to be performed.^12,20^ In this review, we summarize accumulated knowledge on anti-BK virus neutralizing antibodies as well as their clinical importance and therapeutic potential for kidney transplant recipients.

## Anti-BK Virus Neutralization Assays

A standardized and approved assay for the detection of anti-BK virus antibodies is not commercially available. However, different assays, such as hemagglutination inhibition test, ELISA, or multiplex immunoassays, have been developed to determine the presence of BK virus–specific antibodies in patient samples.^21–28^ Most of these serologic assays detect the response directed against the immunodominant VP1 capsid protein because antibodies against the large tumor antigen are infrequent and have low titers, making them unsuitable infection markers in most cases.^29^

The first test to detect anti-BK virus neutralizing antibodies was described in 1986 by Flaegstad *et al.* (Figure 1A).^30^ It was based on plaque reduction detected by immunoperoxidase staining after infection of Vero cells with the Gardner BK virus strain (genotype Ia). Because recombinant BK virus strains are not commercially available for subtypes other than Ia, infectious chimeric viruses encoding genotype II, III, or IV VP1 into a genotype Ia genome were also generated.^31^ Furthermore, as a surrogate model, Christopher Buck's lab developed reporter pseudoviruses on the basis of seven divergent BK virus isolates (Figure 1B).^12,20^ These pseudoviruses are produced *in vitro* by cotransfection of HEK293TT cells with plasmids encoding VP1, VP2, and VP3 capsid proteins as well as a plasmid encoding a reporter protein (typically Luciferase or green fluorescent protein). Although they enter cells like native virions, pseudoviruses cannot replicate and do not propagate infection. Importantly, it has been suggested that neutralization assays offer a greater degree of sensitivity/specificity for serological analysis as compared with ELISA and may better reflect humoral immunity in patients.^20,31^

## BK Virus Serotypes

Using pseudovirions and considering a definition of at least a 100-fold difference in neutralizing titer between two BK virus variants for at least one human serum sample, Pastrana *et al.* demonstrated that BK virus genotypes I, II, III, and IV can be considered as distinct serotypes.^12^ They also wondered whether subgroups Ib1 and Ib2 could be considered as distinct serotypes. However, it is important to note that the Ib2 VP1 sequence they used (PittVR2; accession number: DQ989796) carried two mutations as compared with the Ib2 VP1 consensus (Supplemental Figure 1), that is, E73K and E82D, which are located in the VP1 BC loop (amino acids 57–89) that appears to be a critical determinant for glycan receptor and serotype specificity.^12^ It is thus more reasonable to consider that subtypes Ib1 and Ib2 belong to the same serotype and that these two mutations are responsible for the difference they observed. Similarly, genotype Ic, II, III, IVb1, and IVc2 VP1 sequences used in this study contain, respectively, three, two, five, one, and one mutations as compared with their corresponding consensus, most of which are located in the BC loop. It would therefore be interesting to re-evaluate the number of serotypes using genotype-specific consensus sequences. Nevertheless, this result demonstrates that the difference between two serotypes can be governed by very few residue changes. Interestingly, they also evidenced that each serotype bounds a distinct spectrum of cell surface receptors, suggesting that they have different cellular tropisms and pathogenic potentials *in vivo*.^12^

## BK Virus Neutralizing Antibody Serostatus and Risk of BK Virus–Associated Nephropathy

Using serotype I infectious viruses, Abend *et al.* retrospectively determined the pretransplant BK virus neutralizing antibody serostatus of 116 donor–recipient pairs.^31^ They observed that recipients of kidneys from donors with significant serum neutralizing activity (D+) had elevated risk of BK virus DNAemia, regardless of recipient serostatus (40 D+ versus 76 D−: odds ratio=5.0; 95% confidence interval [CI], 1.9 to 12.7; *P* = 0.0008). Furthermore, donor–recipient pairs with D+/R− neutralizing antibody serostatus had the greatest risk of BK virus DNAemia (odds ratio=4.9; 95% CI, 1.7 to 14.6; *P* = 0.004). They concluded that the presence of BK virus neutralizing antibodies in the donor represents a marker of recent BK virus exposure/replication and possibly a higher viral load in the graft leading to a higher risk of BK virus infection in the recipient. This was in agreement with serology studies and further supported the concept of donor-derived *de novo* BK virus infection that has been postulated by several studies,^32–41^ although an exogenous BK virus infection can also occur. On the other hand, recipient serostatus, regardless of donor serostatus, had no significant effect on the incidence of BK virus DNAemia (31 R+ versus 85 R−: odds ratio=0.83; 95% CI, 0.3 to 2.3; *P* = 0.73), suggesting that the presence of pretransplant BK virus neutralizing antibodies in the recipient does not confer protection against BK virus DNAemia.

However, using pseudoviruses and analyzing sera from 69 donor–recipient pairs, Solis *et al.* observed that genotype mismatch between recipients' neutralization profiles before transplant and their subsequently replicating strain was associated with a significantly higher risk of developing DNAemia (hazard ratio=2.27; 95% CI, 1.06 to 4.88; *P* = 0.04).^14^ In their study, donor neutralizing antibody titers were similar for viruric, viremic, and BK virus–associated nephropathy groups, but a recipient neutralizing antibody titer against the donor's strain lower than 4 log_10_ before transplant was significantly associated with BK virus replication after transplant (hazard ratio=1.88; 95% CI, 1.06 to 3.45; *P* = 0.03). In addition, following 168 kidney transplant recipients, they evidenced an increase of neutralizing antibody titers directed against the replicating strain but also against the other genotypes, probably because of cross-reactivity. Finally, they observed that recipients with high neutralizing antibody titers against the replicating strain at any time had a lower risk of developing BK virus DNAemia (hazard ratio=0.44; 95% CI, 0.26 to 0.73; *P* = 0.002). They thus concluded that the risk of BK virus–associated nephropathy depends on recipient neutralizing antibody kinetics after transplantation rather than donor characteristics.

Nonetheless, the rise of BK virus neutralizing antibody titers may not be the only effector of viral control/clearance. Indeed, Gras *et al.* described 32 patients who experienced BK virus–associated nephropathy despite a strong increase of cross-reactive BK virus neutralizing antibody titers.^42^ In line with these results, Lorentzen *et al.* also described two recipients who received kidneys from the same donor and experienced BK virus–associated nephropathy despite a >1000-fold increase of neutralizing antibody titers: one cleared BK virus DNAemia at 5.5 months after transplant, while the other had persistent BK virus DNAemia of 1.07×10^5^ copies/ml at the last follow-up 52 weeks after transplant.^43^ Furthermore, the crucial role played by BK virus–specific T cells in clearing DNAemia must not be underestimated.^18,44^

## BK Virus Escape from Neutralizing Antibodies

In 2018, Peretti *et al.* showed that, in three patients experiencing BK virus–associated nephropathy, BK virus acquired nonsilent mutations, some of which conferred resistance to neutralization.^34^ By contrast, they did not observe this profile in 23 other patients diagnosed only with BK virus DNAemia. These mutations were suggestive of a role for cellular APOBEC3A/B single-stranded DNA cytosine-to-uracil (C-to-U) deaminases, which were shown to be specifically upregulated by BK virus infection^45,46^ and are known to provide innate antiviral defense by mutating the genomes of DNA-based viruses.^47–49^ They also observed that the mutations were mainly localized in the BC loop of VP1, which is critical for glycan receptor specificity as mentioned above.^50,51^ As a result, they modified the spectrum of glycans engaged for BK virus entry.^34^ These results were further supported by two recent studies from McIlroy *et al.*^52,53^

Alternative mechanisms to evade neutralizing antibodies could also be exploited by BK virus. For instance, *in vitro* experiments suggested that BK virus particles could be internalized by myeloid dendritic cells and transmitted to permissive renal cells through a neutralizing antibody–resistant transinfection mechanism.^54^ BK virus has also been shown to hijack extracellular vesicles for *en bloc* transmission,^55^ and although preliminary results contradict this possibility, this could protect virions from neutralizing antibodies, as described for the JC virus, a related polyomavirus.^56^

## Human Intravenous Igs to Prevent BK Virus–Associated Nephropathy

Studies described above support the potential benefit of administering neutralizing antibodies as a preventive or therapeutic strategy against BK virus infection. In this regard, several groups have considered injection of intravenous Igs (IVIG) to treat kidney allograft recipients with BK virus–associated nephropathy. IVIG is a blood product rich in IgG, derived from pooled human plasma of thousands of donors, which has been shown to be effective in the treatment of acute rejection as well as in the prevention and treatment of viral infections in patients receiving transplant.^57^ Interestingly, several studies confirmed that IVIG preparations contain BK virus reactive antibodies and inhibit all major genotypes at the viral entry step, strongly suggesting that they contain BK virus neutralizing antibodies.^13,15,58–61^ Furthermore, it has been shown that IVIG administration results in a significant increase in BK virus neutralizing antibody titers in kidney transplant recipients, which enables most of them to reach the threshold of 4 log_10_ defined by Solis *et al.*^14,15,62^

The potential benefit of IVIG in the treatment of kidney allograft recipients was first described in 2006.^57^ Eight patients diagnosed with BK virus–associated nephropathy received 2 g/kg IVIG in addition to a reduction of immunosuppressive therapy, and after a mean follow-up of 15 months, 88% kept functioning grafts, although with impaired kidney function. Then, other noncontrolled studies or case reports further supported the fact that IVIG could prevent active BK virus replication in kidney transplant recipients and successfully treat BK virus–associated nephropathy.^63–71^ In a retrospective single-center cohort study, Kable *et al.* also observed that complete or partial clearance of DNAemia was achieved after 3 months in 16 of 28 patients (57.1%) treated by conventional therapy compared with 18 of 22 (81.8%) treated with additional IVIG.^72^ In this study, treatment incorporating IVIG more effectively cleared DNAemia (hazard ratio=3.68; 95% CI, 1.56 to 8.68; *P* = 0.003). More recently, Benotmane *et al.* retrospectively divided kidney transplant recipients into three groups on the basis of neutralizing antibody titers at the day of transplantation: (*1*) patients with neutralizing antibody titers <4 log_10_ who received IVIG for the first 3 months after transplant (*n*=44), (*2*) patients with neutralizing antibody titers <4 log_10_ who did not undergo IVIG treatment (*n*=41), and (*3*) patients with neutralizing antibody titers >4 log_10_ who did not receive IVIG (*n*=89).^62^ They observed that, at 12 months after transplant, the incidence of BK virus DNAemia in the low neutralizing antibody titer group treated with IVIG (6.8%) was markedly lower than that of the low neutralizing antibody titer and IVIG-untreated group (36.6%; *P* < 0.001) and similar to that observed in the high neutralizing antibody titer and IVIG-untreated group (10.1%).

All these studies provide proof-of-concept evidence that IVIG administration could prevent BK virus DNAemia and associated nephropathy. However, they must be interpreted cautiously because of major methodological limitations, such as the lack of a control arm, small sample sizes, heterogeneous conventional therapy and/or IVIG doses (from 2 g/kg divided over 2–5 days to 150 mg/kg every 2 weeks), as well as the concomitant use of other antiviral strategies and/or a parallel reduction of immunosuppression. Some reports also argue against a beneficial use of IVIG in BK virus treatment.^73–76^ Thus, multicentric prospective randomized trials, such as NCT05325008 or NCT04222023 (Table 1), are needed to clearly evaluate the effect of IVIG administration for the prevention or treatment of BK virus DNAemia and associated nephropathy in kidney transplant recipients. NCT05325008 should recruit around 280 participants to compare the efficiency of additional IVIG with immunosuppression reduction/modification in the treatment of kidney or pancreas–kidney transplant recipients with BK virus DNAemia. All participants will be allocated a rank on the basis of all-cause death, allograft loss, eGFR decline, acute allograft rejection or BK virus load higher than 1000 copies/ml, and immunosuppression load at 12 weeks. The primary comparison of interest will be between participants randomized to the IVIG arm as compared with the control arm. On the other hand, NCT04222023 plans to recruit 664 participants to evaluate the effect of IVIG administration for the prevention of BK virus DNAemia in kidney transplant recipients with nondetectable BK virus DNAemia and neutralizing antibody titers lower than 4 log_10_ against the BK virus donor's genotype at the day of transplantation. The incidence of BK virus DNAemia will be evaluated and compared between patients receiving or not IVIG at day 10, 41, and 62 and months 3, 6, and 12. It would also be interesting to screen IVIG batches because the specificities and titers of anti-BK neutralizing antibodies vary depending on supplier.^15^

**Table 1:** Randomized clinical trials evaluating the efficacy of intravenous Igs or monoclonal neutralizing antibodies against BK virus–associated nephropathy

National Clinical Trial Number	Trial Description	Interventions	Recruitment Criteria	Primary Outcome Measures
NCT05325008	Treatment Phase 3 *N*=280 Recruiting	IVIG+Immunosuppression reduction/modification versus immunosuppression reduction/modification	Kidney or pancreas–kidney transplant recipients with BK virus DNAemia (≥5×10^3^ copies/ml)	Composite ordinal outcome on the basis of all-cause death, allograft loss, eGFR decline, acute allograft rejection or BK virus load ≥10^3^ copies/ml, and immunosuppression load
NCT04222023	Prevention Phase 3 *N*=664 Recruiting	IVIG versus no drug	Kidney transplant recipients (including patients receiving multiple-organ transplant) with nondetectable BK virus DNAemia and neutralizing antibody titers ≤4 log_10_ against the BK virus donor's genotype at the day of transplantation	The incidence of BK virus DNAemia (≥10^4^ copies/ml) as measured at days 10, 41, and 62 and months 3, 6, and 12 after transplantation
NCT04294472	Treatment Phase 2 *N*=30 Completed	MAU868 versus placebo	Kidney or pancreas–kidney transplant recipients with BK virus DNAemia (≥10^4^ and ≤10^7^ copies/ml or sustained above 10^3^ copies/ml)	Time (weeks) to decrease of BK virus DNAemia by 1 log_10_ and to first decrease of BK virus DNAemia to less than the lower limit of quantification
NCT05769582	Treatment Phase 2/3 *N*=180 Recruiting	AntiBKV versus placebo	Kidney transplant recipients with BK virus DNAemia (≥10^4^ copies/ml or sustained above 10^3^ copies/ml)	Proportion of participants without detectable BK virus DNAemia at day 141

IVIG, intravenous Igs.

## Monoclonal Neutralizing Antibodies to Prevent BK Virus–Associated Nephropathy

To specifically target BK virus, several teams also generated monoclonal neutralizing antibodies.^77–84^ Most of these monoclonal neutralizing antibodies are expected to interfere with viral binding to its cellular receptor, but it has also been suggested that they could lock together capsid subunits, thereby blocking viral uncoating processes after entry, as proposed for the 41F17/NOV530 monoclonal neutralizing antibody (Supplemental Figure 2).^78,81^

Amplyx Pharmaceuticals and then Vera Therapeutics obtained exclusive global rights from Novartis for one of these monoclonal neutralizing antibodies called MAU868, in 2019 and 2021, respectively. MAU868 is a human IgG1 mAb with high pM binding affinity and sub-nM neutralizing activity against the different BK virus genotypes (EC_50_=0.009–0.093 *μ*g/ml [0.062–0.645 nM]; EC_90_=0.102–4.160 *μ*g/ml [0.708–28.865 nM]).^85^ MAU868 also potently neutralized BK virus variants constructed to contain VP1 sequences from clinical isolates or highly prevalent VP1 polymorphisms as well as JC virus. No resistant variant was identified after serial passage of BK virus in the presence of MAU868 for up to 6 months. The crystal structure of MAU868 in complex with the VP1 pentamer identified a conformational epitope including three contact residues in VP1 that are strictly conserved across BK virus isolates (Y169, R170, K172; Supplemental Figure 2). The safety and efficacy of MAU868 was evaluated in patients receiving kidney transplant with DNAemia in a randomized, double-blind, placebo-controlled phase 2 clinical trial including 20 MAU868 versus eight placebo patients (NCT04294472, Table 1). Patients received four doses of MAU868 (1350 mg ×4 or 6750 mg ×1+1350 mg ×3) or placebo intravenously every 28 days for 12 weeks, with 24 weeks of follow-up. MAU868 was well tolerated and led to rapid and sustained decreases in DNAemia and DNAuria, greater than placebo.^86^ At week 36, 75% of patients having received MAU868 (15/20) showed a DNAemia decrease higher than 1 log_10_ and 30% (6/20) went below the detection limit. By contrast, 50% of controls (4/8) showed a log_10_ DNAemia decrease and none went below the detection limit.

Another monoclonal neutralizing antibody (AntiBKV/MTX-005) developed by Memo Therapeutics recently reached the clinical trial stage. Memo Therapeutics previously patented two monoclonal neutralizing antibodies, 319C07 and 336F07, that broadly neutralize all BK virus serotypes by binding to conformational epitopes (EC_50_=0.007–0.058 and 0.018–0.103 nM for 319C07 and 336F07, respectively).^82^ Binding of 319C07 is affected by N62, D175, or S275 VP1 mutation (Supplemental Figure 2). On the other hand, N62 or E73 VP1 mutations affect 336F07 interaction (Supplemental Figure 2). In contrast to MAU868, these two antibodies do not neutralize the JC virus. AntiBKV has successfully completed a phase 1, single-blind, partially randomized, placebo-controlled study (NCT05358106) aimed at evaluating the safety, tolerability, and pharmacokinetics of single and multiple ascending intravenous doses of the monoclonal neutralizing antibody in healthy adult volunteers. No relevant adverse events were reported in the trial, even with the highest doses, which supported the Fast Track approval by the Food and Drug Administration in May 2023. Memo Therapeutics is currently recruiting patients for a randomized, double-blind, and placebo-controlled phase 2/3 trial (NCT05769582, Table 1). Recipients with BK virus DNAemia will be randomly assigned to receive four doses of AntiBKV by intravenous infusion every 4 weeks or placebo. The primary end point of the trial is the proportion of patients without detectable BK virus in blood at day 92; data are expected by the end of 2024.

Other antibodies with novel structural features and/or improved properties may be patentable in the future.^87^ For instance, in April 2024, SpikImm and SATT Conectus announced the signing of an exclusive option agreement for potent monoclonal antibodies targeting the BK virus, offering a new prophylactic solution for transplant recipients.

## Vaccine Induction of Neutralizing Antibodies to Prevent BK Virus–Associated Nephropathy

Another strategy to protect kidney transplant recipients from BK virus–associated nephropathy would be to administer a vaccine while a patient is on the organ transplant wait list so that high levels of neutralizing antibodies will be present at the time of transplant. This approach was investigated by Christopher Buck, Diana Pastrana, and coworkers at the National Cancer Institute (Bethesda).^12,20,88,89^ To this aim, they produced virus-like particle vaccines similar to approved human papillomavirus vaccines, which are known to induce high levels of neutralizing antibodies and long-lasting protection with an excellent safety record. These virus-like particles spontaneously self-assemble after expression of VP1 in *Escherichia coli*, mammal, baculovirus, or yeast-based expression systems and closely resemble the surface of native polyomavirus virions.^89^ They first showed that mice primed with a single 2*-µ*g dose of a mixture of genotype Ia/Ib1, Ib2, Ic, II, III, IVb1, and IVc2 BK virus-like particles generated in HEK293TT cells yielded neutralizing antibodies against all subtypes, with titers ranging from 3.9 log_10_ (against subtype Ib2) to 4.7 log_10_ (against subtypes IVb1 and IVc2), which was further increased after a booster immunization.^12^ More recently, they also showed that immunization of rhesus macaques with a mixture of 10 *µ*g of BK virus genotype I, II, IV, and JC virus-like particles produced in Sf9 insect cells resulted in neutralizing antibody titers ranging between 2.8 and 6.0 log_10_ after a priming dose, which was slightly increased and maintained for almost 2 years after a booster immunization.^89^ Although these results are encouraging, they need to be confirmed in human clinical trials.

## Conclusion

In the past decade, major progress has been made regarding the understanding of the role played by neutralizing antibodies in prevention of BK virus–associated nephropathy. This could help to anticipate the risk of nephropathy and improve patient monitoring. Recent data also support the potential benefit of administering broadly neutralizing antibodies, such as IVIG or monoclonal neutralizing antibodies, as a therapeutic strategy against BK virus infection. Because of potential escape mutations that accumulate over time, a mixture of neutralizing antibodies recognizing distinct epitopes may be required for optimal therapy. Bispecific antibodies could be used as an alternative strategy but also to broaden the genotype specificity. Besides, other bioengineering approaches on the basis of antibody fragments, such as antigen-binding fragments, single-chain variable fragments, or nanobodies, could improve the stability and solubility of these inhibitors and their bioavailability to the intragraft tubulointerstitial compartment.^90^ In addition, because BK virus DNA can be detected for months in urine and plasma, identification of true replication biomarkers could help to better monitor these therapies.^91^ However, neutralizing antibodies may be more useful before significant BK virus spread and advanced kidney damage, thus as a preventive or preemptive rather than curative strategy. Prospective multicenter randomized trials are highly needed to validate the efficacy of these strategies by overcoming the methodological limitations of proof-of-concept studies and to establish the optimal incorporation of these therapies into current management algorithms. A multivalent BK virus vaccine could also confer protection against BK virus–associated nephropathy and be less expensive, easier to administer, and more durable than neutralizing antibody administration. However, human clinical trials are needed to confirm *in vivo* preclinical results. Such therapeutic strategies are urgently needed considering the scarcity of grafts. Furthermore, their evaluation should include attention not only to clinical benefits but also to cost-effectiveness, taking into account the costs generated by BK virus–related complications. In the near future, all these therapeutic strategies may improve the outcome of not only kidney transplant recipients but also other BK virus–associated diseases, such as hemorrhagic cystitis or bladder cancer.

## Supplementary Material

## Supplemental Material

Supplemental Figure 1. Alignment of the VP1 sequences corresponding to the consensus of the different BK virus subtypes.

Supplemental Figure 2. Mapping of 41F17/NOV530, MAU868, 319C07, and 336F07 monoclonal neutralizing antibody epitopes.
