# Supplementary material for: Neutralizing Antibodies Targeting BK Polyomavirus: Clinical Importance and Therapeutic Potential for Kidney Transplant Recipients
Source: J Am Soc Nephrol. 2024 Jul 9;35(10):1425–33. doi: 10.1681/ASN.0000000000000457 (PMC11452134; doi:10.1681/ASN.0000000000000457)
Supplement: Supplementary file 1 [file jasn-35-1425-s001.pdf]

ASN American Society of Nephrology
JASN Journal of the American Society of Nephrology
KIDNEY360 Accessing Our World From Every Angle
CJASN Clinical Journal of the American Society of Nephrology

ASN Journal Disclosure Form

As per ASN journal policy, I have disclosed any financial relationships or commitments I have held in the past 36 months as included below. I have listed my Current Employer below to indicate there is a relationship requiring disclosure. If no relationship exists, my Current Employer is not listed.

A. Aubry reports the following:
Employer: UPJV - CHU Amiens-Picardie

I understand that the information above will be published within the journal article, if accepted, and that failure to comply and/or to accurately and completely report the potential financial conflicts of interest could lead to the following: 1) Prior to publication, article rejection, or 2) Post-publication, sanctions ranging from, but not limited to, issuing a correction, reporting the inaccurate information to the authors' institution, banning authors from submitting work to ASN journals for varying lengths of time, and/or retraction of the published work.

Name: Aurelien Aubry
Manuscript ID: JASN-2024-000385R1
Manuscript Title: Neutralizing Antibodies Targeting BK Polyomavirus: Clinical Importance and Therapeutic Potential for Kidney Transplant Recipients
Date of Completion: June 18, 2024
Disclosure Updated Date: June 18, 2024

ASN American Society of Nephrology · JASN Journal of the American Society of Nephrology · KIDNEY360 Accessing Our World From Every Angle · CJASN Clinical Journal of the American Society of Nephrology

ASN Journal Disclosure Form

As per ASN journal policy, I have disclosed any financial relationships or commitments I have held in the past 36 months as included below. I have listed my Current Employer below to indicate there is a relationship requiring disclosure. If no relationship exists, my Current Employer is not listed.

E. Brochot has nothing to disclose.

I understand that the information above will be published within the journal article, if accepted, and that failure to comply and/or to accurately and completely report the potential financial conflicts of interest could lead to the following: 1) Prior to publication, article rejection, or 2) Post-publication, sanctions ranging from, but not limited to, issuing a correction, reporting the inaccurate information to the authors' institution, banning authors from submitting work to ASN journals for varying lengths of time, and/or retraction of the published work.

Name: Etienne Brochot
Manuscript ID: JASN-2024-000385R1
Manuscript Title: Neutralizing Antibodies Targeting BK Polyomavirus: Clinical Importance and Therapeutic Potential for Kidney Transplant Recipients
Date of Completion: May 29, 2024
Disclosure Updated Date: May 29, 2024

ASN
American Society of Nephrology
JASN
Journal of the American Society of Nephrology
KIDNEY360
Accessing Our World From Every Angle
CJASN
Clinical Journal of the American Society of Nephrology

ASN Journal Disclosure Form

As per ASN journal policy, I have disclosed any financial relationships or commitments I have held in the past 36 months as included below. I have listed my Current Employer below to indicate there is a relationship requiring disclosure. If no relationship exists, my Current Employer is not listed.

B. Demey has nothing to disclose.

I understand that the information above will be published within the journal article, if accepted, and that failure to comply and/or to accurately and completely report the potential financial conflicts of interest could lead to the following: 1) Prior to publication, article rejection, or 2) Post-publication, sanctions ranging from, but not limited to, issuing a correction, reporting the inaccurate information to the authors' institution, banning authors from submitting work to ASN journals for varying lengths of time, and/or retraction of the published work.

Name: Baptiste Demey
Manuscript ID: JASN-2024-000385R1
Manuscript Title: Neutralizing Antibodies Targeting BK Polyomavirus: Clinical Importance and Therapeutic Potential for Kidney Transplant Recipients,
Date of Completion: June 18, 2024
Disclosure Updated Date: May 29, 2024

ASN American Society of Nephrology | JASN Journal of the American Society of Nephrology | KIDNEY360 Accessing Our World From Every Angle | CJASN Clinical Journal of the American Society of Nephrology

ASN Journal Disclosure Form

As per ASN journal policy, I have disclosed any financial relationships or commitments I have held in the past 36 months as included below. I have listed my Current Employer below to indicate there is a relationship requiring disclosure. If no relationship exists, my Current Employer is not listed.

V. Descamps reports the following:
Employer: CHU AMIENS

I understand that the information above will be published within the journal article, if accepted, and that failure to comply and/or to accurately and completely report the potential financial conflicts of interest could lead to the following: 1) Prior to publication, article rejection, or 2) Post-publication, sanctions ranging from, but not limited to, issuing a correction, reporting the inaccurate information to the authors' institution, banning authors from submitting work to ASN journals for varying lengths of time, and/or retraction of the published work.

Name: Veronique Descamps
Manuscript ID: JASN-2024-000385R2
Manuscript Title: "Neutralizing Antibodies Targeting BK Polyomavirus: Clinical Importance and Therapeutic Potential for Kidney Transplant Recipients,"
Date of Completion: July 2, 2024
Disclosure Updated Date: July 2, 2024

ASN American Society of Nephrology | JASN Journal of the American Society of Nephrology | KIDNEY360 Accessing Our World From Every Angle | CJASN Clinical Journal of the American Society of Nephrology

ASN Journal Disclosure Form

As per ASN journal policy, I have disclosed any financial relationships or commitments I have held in the past 36 months as included below. I have listed my Current Employer below to indicate there is a relationship requiring disclosure. If no relationship exists, my Current Employer is not listed.

F. Helle reports the following:
Employer: Université de Picardie Jules Verne; and Research Funding: Molecular Partners AG.

I understand that the information above will be published within the journal article, if accepted, and that failure to comply and/or to accurately and completely report the potential financial conflicts of interest could lead to the following: 1) Prior to publication, article rejection, or 2) Post-publication, sanctions ranging from, but not limited to, issuing a correction, reporting the inaccurate information to the authors' institution, banning authors from submitting work to ASN journals for varying lengths of time, and/or retraction of the published work.

Name: Francois Helle
Manuscript ID: JASN-2024-000385R1
Manuscript Title: Neutralizing antibodies targeting BK polyomavirus : clinical importance and therapeutic potential for kidney transplant recipients
Date of Completion: May 29, 2024
Disclosure Updated Date: May 29, 2024

ASN American Society of Nephrology | JASN Journal of the American Society of Nephrology | KIDNEY360 Accessing Our World From Every Angle | CJASN Clinical Journal of the American Society of Nephrology

ASN Journal Disclosure Form

As per ASN journal policy, I have disclosed any financial relationships or commitments I have held in the past 36 months as included below. I have listed my Current Employer below to indicate there is a relationship requiring disclosure. If no relationship exists, my Current Employer is not listed.

M. Virginie reports the following:
Employer: AGIR

I understand that the information above will be published within the journal article, if accepted, and that failure to comply and/or to accurately and completely report the potential financial conflicts of interest could lead to the following: 1) Prior to publication, article rejection, or 2) Post-publication, sanctions ranging from, but not limited to, issuing a correction, reporting the inaccurate information to the authors' institution, banning authors from submitting work to ASN journals for varying lengths of time, and/or retraction of the published work.

Name: Morel Virginie
Manuscript ID: JASN-2024-000385R1
Manuscript Title: Neutralizing Antibodies Targeting BK Polyomavirus: Clinical Importance and Therapeutic Potential for Kidney Transplant Recipients
Date of Completion: May 30, 2024
Disclosure Updated Date: May 30, 2024
