# Supplementary material for: Neutralizing Antibodies Targeting BK Polyomavirus: Clinical Importance and Therapeutic Potential for Kidney Transplant Recipients
Source: J Am Soc Nephrol. 2024 Jul 9;35(10):1425–33. doi: 10.1681/ASN.0000000000000457 (PMC11452134; doi:10.1681/ASN.0000000000000457)
Supplement: Supplementary file 2 [file jasn-35-1425-s002.pdf]

Supplemental Material table of contents

Supplemental Figure 1: Alignment of the VP1 sequences corresponding to the consensus of the different BK virus subtypes.

Supplemental Figure 2: Mapping of 41F17 / NOV530, MAU868, 319C07 and 336F07 monoclonal neutralizing antibody epitopes.

```
                                                                                          ←——— BC-loop ———→
Ia      MAPTKRKGEC PGAAPKKPKE PVQVPKLLIK GGVEVLEVKT GVDAITEVEC FLNPEMGDPD ENLRGFSLKL SAENDFSSDS PERKMLPCYS   90
Ib1     MAPTKRKGEC PGAAPKKPKE PVQVPKLLIK GGVEVLEVKT GVDAITEVEC FLNPEMGDPD ENLRGFSLKL SAENDFSSDS PERKMLPCYS   90
Ib2     MAPTKRKGEC PGAAPKKPKE PVQVPKLLIK GGVEVLEVKT GLDAITEVEC FLNPEMGDPD ENLRGFSLKL SAENDFSSDS PERKMLPCYS   90
Ic      MAPTKRKGEC PGAAPKKPKD PVQVPKLLIK GGVEVLEVKT GVDAITEVEC FLNPEMGDPD ENLRGFSLKL SAENDFSSDS PERKMLPCYS   90
II      MAPTKRKGEC PGAAPKKPKE PVQVPKLLIK GGVEVLEVKT GVDAITEVEC FLNPEMGDPD DNLRGYSLKL TAENAFDSDS PDKKMLPCYS   90
III     MAPTKRKGEC PGAAPKKPKE PVQVPKLLIK GGVEVLEVKT GVDAITEVEC FLNPEMGDPD DNLRGYSQHL SAENAFESDS PDRKMLPCYS   90
IVa1    MAPTKRKGEC PGAAPKKPKE PVQVPKLLIK GGVEVLEVKT GVDAITEVEC FLNPEMGDPD NDLRGYSLRL TAETAFESDS PDRKMLPCYS   90
IVa2    MAPTKRKGEC PGAAPKKPKD PVQVPKLLIK GGVEVLEVKT GVDAITEVEC FLNPEMGDPD NDLRGYSLRL TAETAFDSDS PDRKMLPCYS   90
IVb1    MAPTKRKGEC PGAAPKKPKE PVQVPKLLIK GGVEVLEVKT GVDAITEVEC FLNPEMGDPD NDLRGYSLRL TAETAFDSDS PDRKMLPCYS   90
IVb2    MAPTKRKGEC PGAAPKKPKE PVQVPKLLIK GGVEVLEVKT GVDAITEVEC FLNPEMGDPD NDLRGYSLRL TAETAFDSDS PDRKMLPCYS   90
IVc1    MAPTKRKGEC PGAAPKKPKE PVQVPKLLIK GGVEVLEVKT GVDAITEVEC FLNPEMGDPD NDLRGYSLRL TAETAFDSDS PDRKMLPCYS   90
IVc2    MAPTKRKGEC PGAAPKKPKE PVQVPKLLIK GGVEVLEVKT GVDAITEVEC FLNPEMGDPD NDLRGYSLRL TAETAFDSDS PDRKMLPCYS   90

Ia      TARIPLPNLN EDLTCGNLLM WEAVTVQTEV IGITSMLNLH AGSQKVHEHG GGKPIQGSNF HFFAVGGDPL EMQGVLMNYR TKYPDGTITP  180
Ib1     TARIPLPNLN EDLTCGNLLM WEAVTVQTEV IGITSMLNLH AGSQKVHEHG GGKPIQGSNF HFFAVGGDPL EMQGVLMNYR TKYPDGTITP  180
Ib2     TARIPLPNLN EDLTCGNLLM WEAVTVQTEV IGITSMLNLH AGSQKVHEHG GGKPIQGSNF HFFAVGGDPL EMQGVLMNYR TKYPEGTITP  180
Ic      TARIPLPNLN EDLTCGNLLM WEAVTVQTEV IGITSMLNLH AGSQKVHEHG GGKPIQGSNF HFFAVGGDPL EMQGVLMNYR TKYPEGTITP  180
II      TARIPLPNLN EDLTCGNLLM WEAVTVKTEV IGITSMLNLH AGSQKVHENG GGKPVQGSNF HFFAVGGDPL EMQGVLMNYR TKYPQGTITP  180
III     TARIPLPNLN EDLTCGNLLM WEAVTVKTEV IGITSMLNLH AGSQKVHENG GGKPVQGSNF HFFAVGGDPL EMQGVLMNYR TKYPQGTITP  180
IVa1    TARIPLPNLN EDLTCGNLLM WEAVTVKTEV IGITSMLNLH AGSQKVHENG GGKPIQGSNF HFFAVGGDPL EMQGVLMNYR TKYPEGTVTP  180
IVa2    TARIPLPNLN EDLTCGNLLM WEAVTVKTEV IGITSMLNLH AGSQKVHENG GGKPIQGSNF HFFAVGGDPL EMQGVLMNYR TKYPEGTVTP  180
IVb1    TARIPLPNLN EDLTCGNLLM WEAVTVKTEV IGITSMLNLH AGSQKVHDNG GGKPIQGSNF HFFAVGGDPL EMQGVLMNYR TKYPEGTVTP  180
IVb2    TARIPLPNLN EDLTCGNLLM WEAVTVKTEV MGITSMLNLH AGSQKVHDNG GGKPIQGSNF HFFAVGGDPL EMQGVLMNYR TKYPEGTVTP  180
IVc1    TARIPLPNLN EDLTCGNLLM WEAVTVKTEV IGITSMLNLH AGSQKVHENG GGKPVQGSNF HFFAVGGDPL EMQGVLMNYR TKYPEGTVTP  180
IVc2    TARIPLPNLN EDLTCGNLLM WEAVTVKTEV IGITSMLNLH AGSQKVHENG GGKPIQGSNF HFFAVGGDPL EMQGVLMNYR TKYPEGTVTP  180

Ia      KNPTAQSQVM NTDHKAYLDK NNAYPVECWV PDPSRNENTR YFGTFTGGEN VPPVLHVTNT ATTVLLDEQG VGPLCKADSL YVSAADICGL  270
Ib1     KNPTAQSQVM NTDHKAYLDK NNAYPVECWV PDPSRNENTR YFGTFTGGEN VPPVLHVTNT ATTVLLDEQG VGPLCKADSL YVSAADICGL  270
Ib2     KNPTAQSQVM NTDHKAYLDK NNAYPVECWI PDPSRNENTR YFGTFTGGEN VPPVLHVTNT ATTVLLDEQG VGPLCKADSL YVSAADICGL  270
Ic      KNPTAQSQVM NTDHKAYLDK NNAYPVECWI PDPSRNENTR YFGTLTGGEN VPPVLHVTNT ATTVLLDEQG VGPLCKADSL YVSAADICGL  270
II      KNPTAQSQVM NTDHKAYLDK NNAYPVECWI PDPSRNENTR YFGTYTGGEN VPPVLHVTNT ATTVLLDEQG VGPLCKADSL YVSAADICGL  270
III     KNPTAQSQVM NTDHKAYLDK NNAYPVECWI PDPSRNENTR YFGTYTGGEN VPPVLHVTNT ATTVLLDEQG VGPLCKADSL YVSAADICGL  270
IVa1    KNPTAQSQVM NTDHKAYLDK NNAYPVECWI PDPSRNENTR YFGTYTGGEN VPPVLHVTNT ATTVLLDEQG VGPLCKADSL YVSAADICGL  270
IVa2    KNPTAQSQVM NTDHKAYLDK NNAYPVECWI PDPSRNENTR YFGTYTGGEN VPPVLHVTNT ATTVLLDEQG VGPLCKADSL YVSAADICGL  270
IVb1    KNPTAQSQVM NTDHKAYLDK NNAYPVECWI PDPSRNENTR YFGTYTGGEN VPPVLHVTNT ATTVLLDEQG VGPLCKADSL YVSAADICGL  270
IVb2    KNPTAQSQVM NTDHKAYLDK NNAYPVECWI PDPSRNENTR YFGTYTGGEN VPPVLHVTNT ATTVLLDEQG VGPLCKADSL YVSAADICGL  270
IVc1    KNPTAQSQVM NTDHKAYLDK NNAYPVECWI PDPSRNENTR YFGTYTGGEN VPPVLHVTNT ATTVLLDEQG VGPLCKADSL YVSAADICGL  270
IVc2    KNPTAQSQVM NTDHKAYLDK NNAYPVECWI PDPSRNENTR YFGTYTGGEN VPPVLHVTNT ATTVLLDEQG VGPLCKADSL YVSAADICGL  270

Ia      FTNSSGTQQW RGLARYFKIR LRKRSVKNPY PISFLLSDLI NRRTQRVDGQ PMYGMESQVE EVRVFDGTER LPGDPDMIRY IDKQGQLQTKML 362
Ib1     FTNSSGTQQW RGLARYFKIR LRKRSVKNPY PISFLLSDLI NRRTQRVDGQ PMYGMESQVE EVRVFDGTER LPGDPDMIRY IDKQGQLQTKML 362
Ib2     FTNSSGTQQW RGLARYFKIR LRKRSVKNPY PISFLLSDLI NRRTQRVDGQ PMYGMESQVE EVRVFDGTER LPGDPDMIRY IDKQGQLQTKML 362
Ic      FTNSSGTQQW RGLARYFKIR LRKRSVKNPY PISFLLSDLI NRRTQRVDGQ PMYGMESQVE EVRVFDGTEK LPGDPDMIRY IDKQGQLQTKML 362
II      FTNSSGTQQW RGLARYFKIR LRKRSVKNPY PISFLLSDLI NRRTQKVDGQ PMYGMESQVE EVRVFDGTEQ LPGDPDMIRY IDRQGQLQTKMV 362
III     FTNSSGTQQW RGLARYFKIR LRKRSVKNPY PISFLLSDLI NRRTQKVDGQ PMYGMESQVE EVRVFDGTEQ LPGDPDMIRY IDRQGQLQTKMV 362
IVa1    FTNSSGTQQW RGLPRYFKIR LRKRSVKNPY PISFLLSDLI NRRTQRVDGQ PMYGMESQVE EVRVFDGTEQ LPGDPDMIRY IDRQGQLQTKMV 362
IVa2    FTNSSGTQQW RGLPRYFKIR LRKRSVKNPY PISFLLSDLI NRRTQRVDGQ PMYGMESQVE EVRVFDGTEQ LPGDPDMIRY IDRQGQLQTKMV 362
IVb1    FTNSSGTQQW RGLPRYFKIR LRKRSVKNPY PISFLLSDLI NRRTQRVDGQ PMYGMESQVE EVRVFDGTEK LPGDPDMIRY IDRQGQLQTKMV 362
IVb2    FTNSSGTQQW RGLPRYFKIR LRKRSVKNPY PISFLLSDLI NRRTQRVDGQ PMYGMESQVE EVRVFDGTEQ LPGDPDMIRY IDRQGQLQTKMV 362
IVc1    FTNSSGTQQW RGLPRYFKIR LRKRSVKNPY PISFLLSDLI NRRTQRVDGQ PMYGMESQVE EVRVFDGTEQ LPGDPDMIRY IDRQGQLQTKMV 362
IVc2    FTNSSGTQQW RGLPRYFKIR LRKRSVKNPY PISFLLSDLI NRRTQRVDGQ PMYGMESQVE EVRVFDGTEQ LPGDPDMIRY IDRQGQLQTKMV 362
```

Supplemental Figure 1 : Alignment of the VP1 sequences corresponding to the consensus of the different BK virus subtypes. The consensus sequences were established with the sequences described in [9] using Simple Consensus Maker (https://www.hiv.lanl.gov/content/sequence/CONSENSUS/SimpCon.html). Amino acids that vary between subtypes are highlighted in yellow. The BC-loop (amino acids 57-89), a critical determinant for glycan receptor and serotype specificity, is indicated in red.

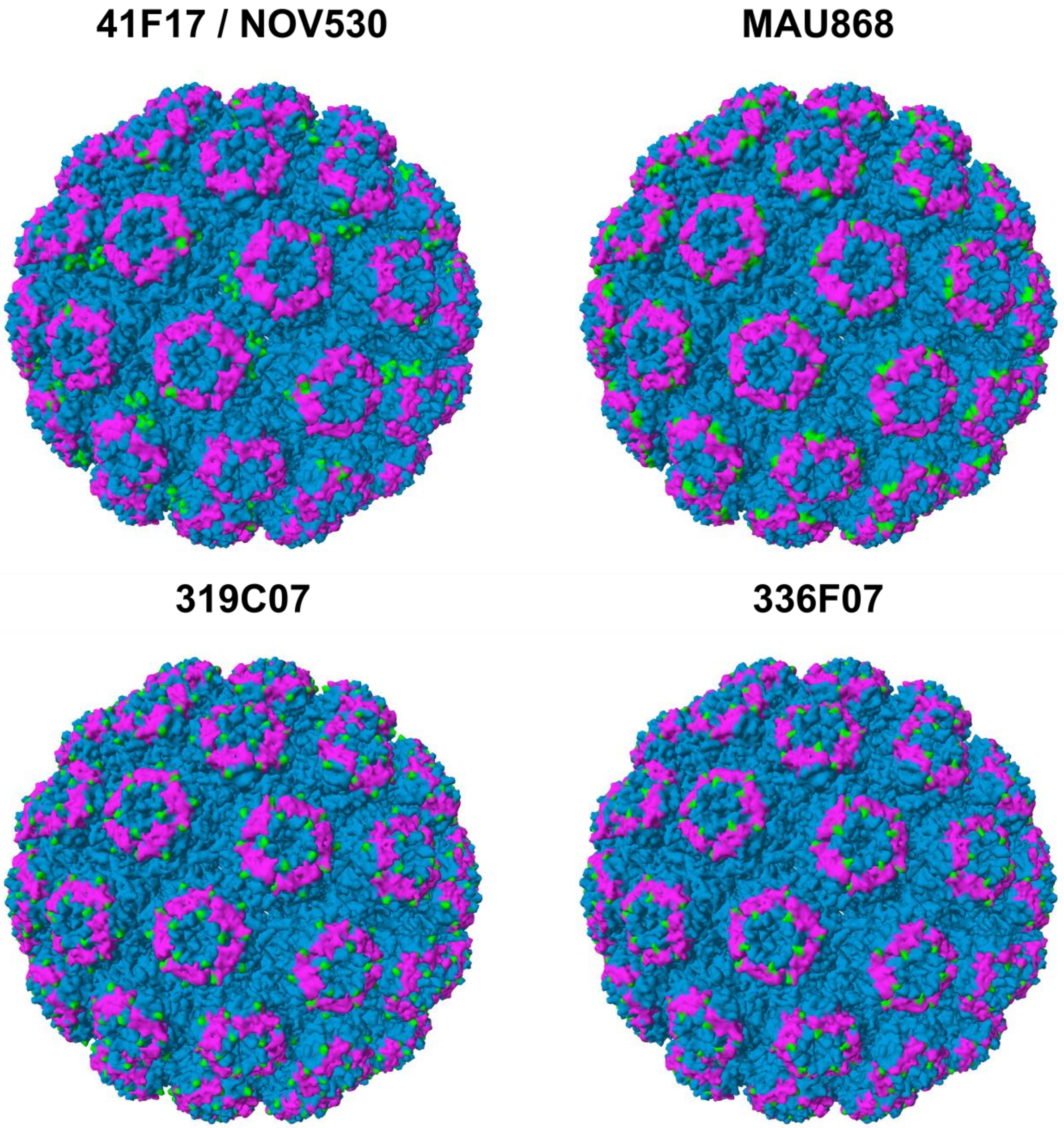

Supplemental Figure 2 : Mapping of 41F17 / NOV530, MAU868, 319C07 and 336F07 monoclonal neutralizing antibody epitopes. Images were created using Mol* Viewer [92] and obtained from the Research Collaboratory for Structural Bioinformatics Protein Data Bank (rcsb.org) [93] (PDB ID: 6ESB [7]). The BC-loop (amino acids 57-89), a critical determinant for glycan receptor and serotype specificity, is highlighted in pink. The amino acids potentially involved in the binding of each antibody are highlighted in light green (D318/P321/Y323/M325, Y169/N182/T184/Q186/N191/T192 and P59/D60/R64/G176/K200 in three different VP1 monomers for 41F17 / NOV530 [78,81] ; Y169, R170 and K172 for MAU868 [85] ; N62, D175 and S275 for 319C07 [82] ; N62 and E73 for 336F07 [82]).
